# Sensoring Fusion Data from the Optic and Acoustic Emissions of Electric Arcs in the GMAW-S Process for Welding Quality Assessment

**DOI:** 10.3390/s120606953

**Published:** 2012-05-25

**Authors:** Sadek Crisóstomo Absi Alfaro, Eber Huanca Cayo

**Affiliations:** Automation and Control Group in Manufacturing Processes—GRACO, University of Brasilia, Faculty of Technology, Department of Mechanical, Mechatronic Engineering, Campus Universitario Darcy Ribeiro, Asa Norte, 70910-900-Brasilia/DF, Brazil; E-Mail: eber@unb.br

**Keywords:** data fusion, arc emissions, quality, GMAW-S

## Abstract

The present study shows the relationship between welding quality and optical-acoustic emissions from electric arcs, during welding runs, in the GMAW-S process. Bead on plate welding tests was carried out with pre-set parameters chosen from manufacturing standards. During the welding runs interferences were induced on the welding path using paint, grease or gas faults. In each welding run arc voltage, welding current, infrared and acoustic emission values were acquired and parameters such as arc power, acoustic peaks rate and infrared radiation rate computed. Data fusion algorithms were developed by assessing known welding quality parameters from arc emissions. These algorithms have showed better responses when they are based on more than just one sensor. Finally, it was concluded that there is a close relation between arc emissions and quality in welding and it can be measured from arc emissions sensing and data fusion algorithms.

## 1. Introduction

Gas metal arc welding—GMAW in short circuit transfer mode (GMAW-S), is a manufacturing process widely used in the metallic construction industry. Diverse advantages such as the high rate of metal transference, elevated penetration and facility for welding in diverse positions, makes this process the most widely used. One way of defining welding quality is through standard specifications which list the limits of discontinuities which are acceptable for a particular application. Quality specifications are not the same for all weld applications. A weld acceptable for static loading may not be acceptable for a dynamic loading application. Six items for assuring the weld quality must be considered: process selection, joint penetration, procedure, pretesting, qualified personnel and in-process monitoring [1]. The present work focuses on in-process monitoring of weld quality.

Many efforts have been encouraged by the industry in order to guarantee welding quality. One of them is the on-line monitoring of some welding parameters which reduces the severity and time requirements of the quality control tests. Classically, the arc tension and welding current are monitored. These parameters are electric arc stability indicators and their behavior also has direct implications in the heat and metal transference which is reflected in the weld bead geometry. High stability in welding does not necessarily mean high quality. Welding quality, in addition to stability, involves other requirements according its application, but certainly the stability is an essential condition. Beside voltage and current parameters, during arc welding operations, the electric arc produces electromagnetic and mechanical emissions observed as magnetic fields, luminescence, light flashing and sound (named as arc emissions). Typically welders use these emissions in combination with their knowledge as feedback information for controlling the welding process aiming to achieve high quality. Different researches shows that is possible to detect some interferences and assess the welding quality by measuring acoustic and optical arc emissions [2–10]. There is an absolute dependence of acoustic emissions coming from arc welding for controlling the process in manual welding operations [5]. The welders “pay attention” basically to the stationarity of the sound signal during welding. This signal is very reliable when the delay is not great than 400 ms. Beside acoustic emissions, the welding arc also generates electromagnetic emissions and certainly, the welder also uses this information in form of an image of the welding pool and its brightness behavior for controlling the welding process [5]. In that case, the continuity of the welding pool image format and its brightness are also desirable for assuring the welding quality. It was noticed that in past research works, the arc emissions were processed separately. A processing method based on a combination of acoustic and electromagnetic emissions (data fusion) could yield interesting information about arc emissions. The goal of this paper is to show the performance of a known data fusion model for specifically assessing welding quality by monitoring its arc emissions. The welding quality assessment using sensoring of the arc emissions could allow detecting disturbances that originate defects in weld beads.

### 1.1. Arc Emissions

**Figure 1.**
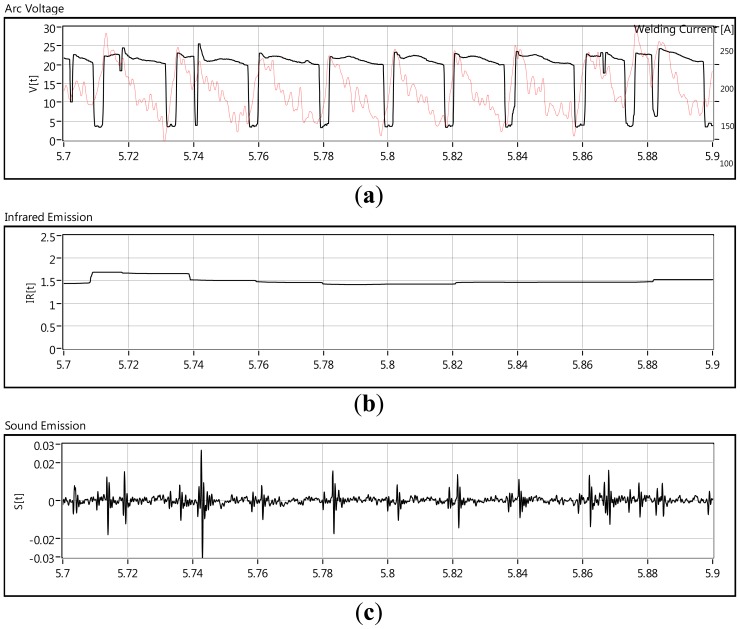
Welding arc parameters and emissions.

The electric arc is a current flowing between two electrodes through an ionized column of gas called a plasma. The space between the two electrodes can be divided into three areas of heat generation: the anode, the cathode and the arc plasma [11]. In the welding arc the electrons flow from cathode to anode and the positive ions flow from anode to cathode. These have been accelerated through the plasma by the arc voltage and they give up their energy as heat. The heat is generated in the cathode area mostly by the positive ions striking the surface of the cathode as well as the heat is generated at the anode mostly by the electrons. These electrons, atoms and ions that are flowing along the plasma column are in accelerated motion and constantly colliding. This chaotic flow together with the heat and the electromagnetic fields of the welding arc produces the arc emissions of electromagnetic nature such as the infrared emission. Besides electromagnetic emissions, the welding arc produces acoustic emissions, principally due to changes in the electric power in the arc column [3]. Figure 1 shows a waveform chart of GMAW-S process parameters monitored. simultaneously They are: arc voltage and welding current (a), the infrared emission (b) and the acoustic emission (c).

#### 1.1.1. Infrared Emission

Infrared emission is originated by the electromagnetic energy emitted by the welding arc and sensed just at the infrared wavelength (0.8–1.1 μm specified in the pyrometer datasheet). Its intensity and wavelength of energy produced depends on the welding parameters, electrode and base metal composition, as well as the fluxes of shielding gas. The intensity of this electromagnetic emission I_e is governed by Planck's law which describes the spectral radiance of unpolarized electromagnetic radiation at all wavelengths emitted from a black body at absolute temperature T. As a function of frequency v, Planck's law is written as:

(1) $$I_{e}\left(v,T\right)=\frac{2hv^{3}}{c^{2}}\frac{1}{e^{\left[\frac{hv}{kT}\right]}-1}$$

In Equation (1), I_e_ is also named as spectral radiance (jm^2^sr^−1^), T temperature (k), v frequency (HZ), h Plank constant (6.62606896 × 10^−34^ Js), c speed of light (3.0 × 10^8^m/s) and k Boltzmann constant (≈1.3806504 × 10^−23^)J/k).

Figure 1(b) shows the infrared radiation response and as it can be seen, infrared emissions do not match the arc voltage and welding current behavior (see Figure 1(a)), but by monitoring IR emissions, it is possible to monitor features such as bead width and penetration [1,12,13]. In the next section it will be shown that the infrared radiation has a direct relation with the welding arc power.

#### 1.1.2. Sound Emission

In the GMAW-S process, the metal is transferred to the welding pool when the molten tip of the consumable electrode contacts the molten puddle. This generates sudden changes in the power of the welding arc. In GMAW-S, the welding arc is characterized by ignitions and extinction sequences and the welding arc sound fits this welding arc behavior. In each arc ignition there is a sound peak as well as when the arc has been extinct, a small sound peak is produced (see Figures 1(a,c)). It is also noticed that there is a delay in the sound compared with the arc voltage signals; this delay is produced by the airborne nature of the acoustic emission [2]. The correspondence between the welding arc sound emission S_e_(t) and the welding arc power P(t)=V(t) I(t)could be expressed by Equation (2).

(2) $$S_{e}\left(t\right)\approx K\left[\frac{d\left(P\left(t\right)\right)}{dt}\right]$$

(3) $$k=\alpha \left(\gamma -1\right)/c^{2}$$

where K is a proportionality factor, α is a geometrical factor, γ the adiabatic expansion coefficient of air and c the velocity of sound in the arc (Equation (3)).

### 1.2. Stationarity of Arc Emissions

Stationarity is a statistical property of random nature signals which means that the statistical quantities are independent of the absolute time and dependant only on relative times, in other words a signal is stationarity when its essential statistical properties are invariant over time. Two kinds of stationarity are distinguished: weak and strong stationarity. Weak stationarity is meant when the first and second moments are independent of time and constants, that is, 〈E_t_〉 = μ and 〈| E_t_ − μ |^2^〉= σ ^2^, (where 〈 〉 stands for the ensemble average). For finite random signals that is the case of the welding arc emissions, the behavior of the mean value and variance cannot be enough estimators for stationarity. A stochastic process {E_t_} with t as an integer number, is denominated as strongly stationary if any set of times t_1_,t_2_ and any integer k the joint probability distributions of $\left\{{\text{E}}_{{\text{t}}_{1}}{,\ldots ,\text{E}}_{{\text{t}}_{\text{n}}}\right\}$ and $\left\{E_{t_{1+k}},\ldots ,E_{t_{n+k}}\right\}$coincide, in other words, when there is correlation between both distributions. Before to calculate the autocorrelation function is necessary obtain some statistical parameters considering each arc emission E(t,β) as a stochastic variable.

Probability average:

(4) $$\begin{array}{c} {\langle \text{E}\rangle }_{\text{j}}=\underset{\text{N}\to \infty }{\lim}\frac{1}{\text{N}}\left[\sum_{\text{i}=1}^{\text{N}}\text{E}\left(\beta_{\text{i}},\tau_{\text{j}}\right)\right]\\ \text{j}=1,2,\ldots ,\text{M}+1 \end{array}$$

where N is the number of realizations of the process M is the number of time steps and β is the random variable.

Time Average:

(5) $$\bar{\text{E}}=\underset{\text{T}\to \infty }{\lim}\frac{1}{2\text{T}}\int_{0}^{\text{T}}\text{E}\left(\text{t}\right)\text{dt}$$

Fluctuations:

(6) $${\text{E}}^{\prime }(t)=\text{E}\left(\text{t}\right)-\bar{\text{E}}$$

Since $\bar{E^{\prime }\left(\text{t}\right)}=0$, the variance is simply calculated as:

(7) $$\sigma_{S^{\prime }}^{2}=\bar{{{\text{E}}^{\prime }}^{2}}$$

The time average of the square of the fluctuations is evaluated by using Equation (8).

(8) $$\bar{{{\text{E}}^{\prime }}^{2}}=\underset{\text{T}\to \infty }{\lim}\frac{1}{\text{N}}\int_{0}^{\text{T}}{{\text{E}}^{\prime }}^{2}\left(\text{t}\right)\text{dt}$$

Finally the autocorrelation is defined as:

(9) RE′(τ)=〈E'(t+τ)E'(t)〉=E'(t+τ)E'(t)¯

It is more convenient to work with the normalized autocorrelation function AcF_E′_ defined in Equation (10). Note that AcF_E′_ =1 indicates weak stationarity and AcF_E′_ =0 indicates strong stationarity

(10) $${\text{ACF}}_{{\text{E}}^{\prime }}\left(\tau \right)=\frac{{\text{R}}_{{\text{E}}^{\prime }}\left(\tau \right)}{\sqrt{\bar{{{\text{E}}^{\prime }}^{2}\left(\text{t}\right)}}\sqrt{{{\text{E}}^{\prime }}^{2}\left(\text{t}+\tau \right)}}$$

**Figure 2.**
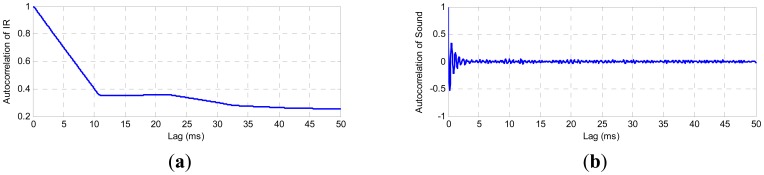
Welding arc emissions autocorrelation.

Note that c_E′_ = 1 indicates weak stationarity and c_E′_ = 0 indicates strong stationarity. Figure 2(a,b) show plots of the normalized autocorrelation of sound and infrared emissions.

Generally, the autocorrelation is expected to decay exponentially, and the fluctuations are expected to become uncorrelated after a sufficiently long-time. In the above figures it is observed that autocorrelation functions tend to zero, which means that both welding arc emissions have a strong stationarity after a certain time and therefore they can be used as welding monitoring parameters.

## 2. Experimental Setup

**Figure 3.**
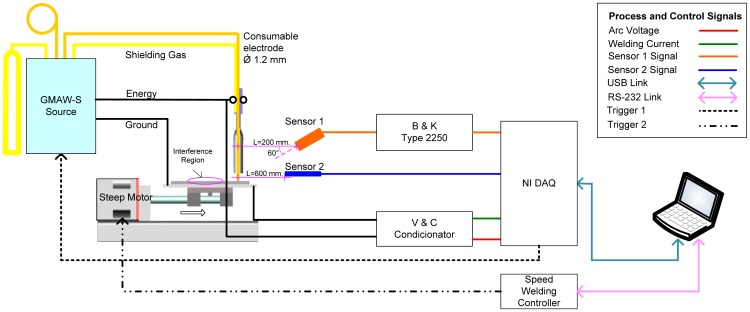
Experimental Setup.

Figure 3 shows the experimental apparatus, consisting of a welding power source, sensoring equipment, data acquisition card and virtual instrumentation software used for data acquisition based on arc voltage, welding current and arc emissions signals. Those signals were sampled at 20 kHz. Arc voltage was acquired by a voltage shunt and optical insulator connected to the acquisition card. The welding current was acquired by a Hall Effect sensor linked at acquisition card previously conditioned. Arc emissions sensoring details are shown in Figure 3. The first sensor is the decibel meter B&K 2250, it uses a 4189 type microphone with −26 ± 1.5 dB gain, ±1.0 output amplitude signal and sensitivity of 50 V/Pa. This device was covered with an aluminum shell for protection against welding spatter and was positioned at 200 mm from the arc. Its output was linked to decibel meter B&K 2,250 whose output (±5 V) finally was linked to acquisition card. The second sensor is the TL-S-25pyrometer is housed inside a stainless steel shield; its measuring output signal is the standard loop current 4–20 mA which is proportional to monitored temperature. This sensor was located at 600 mm. from welding pool following its technical recommendations.

The welds were carried out on steel plates AISI 1020 (140 mm × 101.2 × 9.60 mm) using AWS A5.18 ER70S-6 1.2 mm in diameter electrode wire; the shield gas was the mixture of argon and carbonic anhydride M21 (ATAL 5A/Ar 82% + CO_2_ 18%). The welding runs were performed maintain a fixed contact tip work distance—CTWD at 10 mm and shield gas flow at 15 L/min. These experiments were executed setting combinations for four arc voltage levels (18, 19, 20 and 21 V), five levels to wire feed speed (3.0, 3.5, 4.0, 4.5 and 5.0 m/min) and three welding speed levels (7, 9 and 11 mm/s) which in total gives sixty welding experiments.

## 3. Results and Discussion

### 3.1. Assessment System

**Figure 4.**
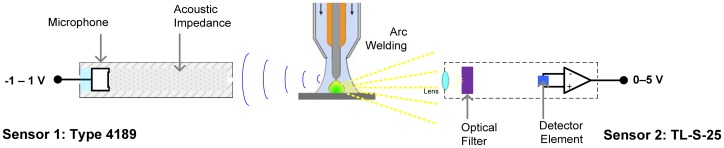
Competitive topology.

In data fusion theory, there are three principal architecture topologies that are categorized according to the type of sensor configurations: complementary, competitive and cooperative. In this work, the competitive topology was used (see Figure 4). In this type of configuration, each sensor (acoustic and electromagnetic) delivers independent measurements of the same attribute or feature (welding arc behavior).

**Figure 5.**
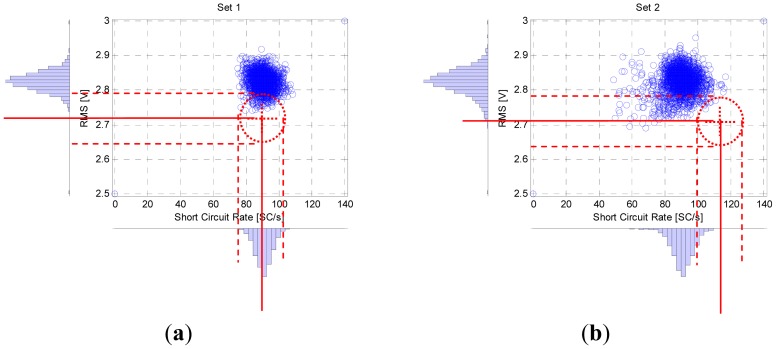

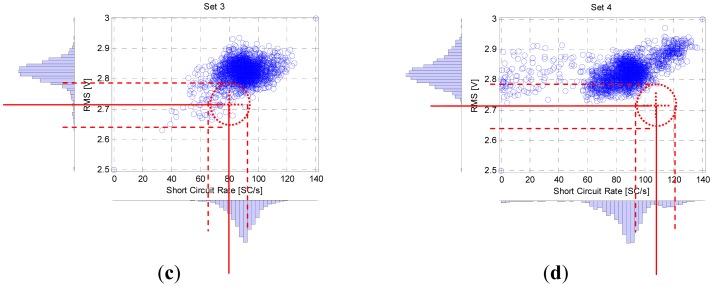
Distribution of RMS and Short circuit rate signal.

Figure 5 shows the simultaneous statistic distributions (probability density distribution—PDD) of the RMS of IR emission and the short circuit rate from acoustic correspondent to experiments: free disturbances (Figure 5(a)), CTWD variation (Figure 5(b)), grease on plate (Figure 5(c)) and lack of shielding gas supply (Figure 5(d)). The PDDs of the monitored parameter for the free disturbances experiments (Figure 5(a)) show a normal distribution, the fine line represent the average and the dotted lines represent the three times of standard deviation. On each distribution, there is an elliptic region that indicates approximately the stability zone which means that the welding has a high quality while IgR and RMS parameters are inside this zone. This ellipse is centered on the intersection of the averages of profile parameters. The proportion of the axes of ellipse defines the stability zone and they are determined by the Equations (11–12).

These set equations are based on third deviation rule; the expression $|{\bar{\text{P}}}_{\text{i}}^{\text{k}}-\bar{{\text{P}}^{\text{k}}}|/3\bar{{\text{S}}_{\text{k}}}$ represents the distance of Mahalanobis, what indicates the difference between the value of average reference and the measured value related with the three times the standard deviation (99.7% of confidence). In theoretical case if the measured value is equal to average value, the quality of the measured value is the best. In practice this value is hardly reached. For quality assessment the difference between the unity and the Mahalanobis distance (Equation (1)) was expressed in percent terms (from 0 to 100%) corresponding 0% to lowest quality as well as 100% to the best.

(11) $${\bar{\text{P}}}_{\text{i}}^{\text{k}}=\frac{1}{\text{n}}\sum_{\text{i}=1}^{\text{n}}{\text{P}}_{\text{i}}^{\text{k}}=\frac{1}{\text{n}}\left({\text{P}}_{1}^{\text{k}}+\cdots {\text{P}}_{\text{n}}^{\text{k}}\right)$$

(12) $${\text{QL}}_{\text{i}}^{\text{k}}=\{\begin{array}{cc} 0\% & \left|{\bar{\text{P}}}_{\text{i}}^{\text{k}}-\bar{{\text{P}}^{\text{k}}}\right|>3\bar{{\text{S}}_{\text{k}}}\\ \left[1-\frac{\left|{\bar{\text{P}}}_{\text{i}}^{\text{k}}-\bar{{\text{P}}^{\text{k}}}\right|}{3\bar{{\text{S}}_{\text{k}}}}\right].100100\%, & 0<\left|{\bar{\text{P}}}_{\text{i}}^{\text{k}}-\bar{{\text{P}}^{\text{k}}}\right|\leq 3\bar{{\text{S}}_{\text{K}}} \end{array}$$

where ${\bar{\text{P}}}_{\text{i}}^{\text{k}}$ is the average of the k assessed parameter on the i moving window, ${\text{P}}_{\text{i}}^{\text{k}}$ sample data from k parameter, n data window size, $\bar{{\text{P}}^{\text{K}}}$ reference average of the k assessed parameter, $\bar{{\text{S}}_{\text{k}}}$ reference standard deviation of the k assessed parameter, ${\text{QL}}_{\text{i}}^{\text{k}}$ quality level for assessed parameter k on the moving window i.

**Figure 6.**
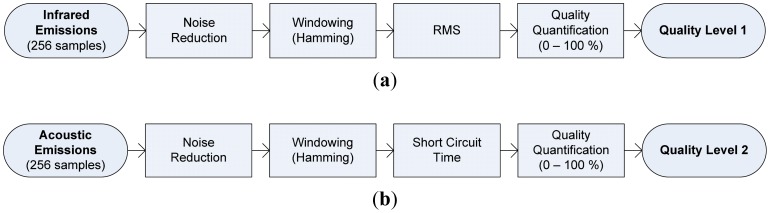
Pre-processing data signal stages, (**a**) infrared signal, (**b**) acoustical signal.

**Figure 7.**
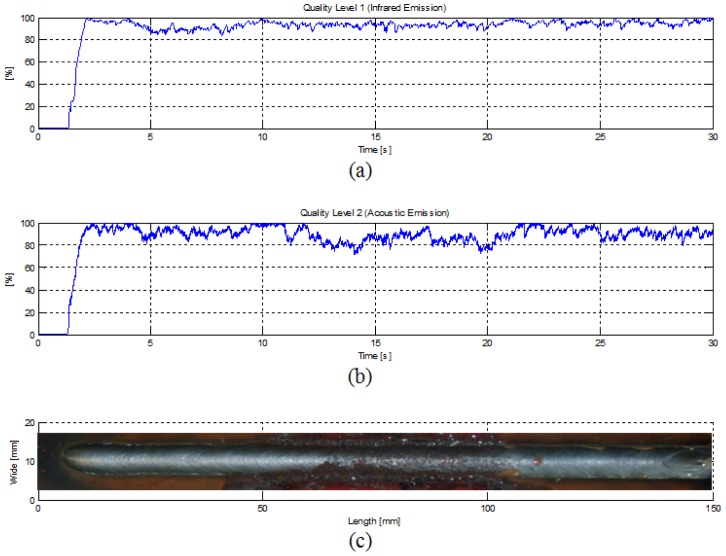
Quality level parameters, (**a**) From infrared signal, (**b**) From acoustical signal, (**c**) welding trial with induced perturbation.

Figure 6 shows the different pre-processing stages applied at each signal sensor. Data signal segments of 256 samples are pre-processed with a overlap of 75%; a noise reduction stage is performed, before hamming windowing. In the case of infrared emission signals, the root mean square—RMS was extracted for each data window, with this result, the welding quality quantification stage was carried out. In the case of the acoustic signals, the short circuit time measured and assessed by the quality quantification stage. The quality quantification for both signals were performed based on statistical control process rules described by the Equations (11) and (12) applied at each data windows. This data processing sequence was carried out for each sensor as is shown in Figure 7(a,b).

In Figure 7(a,b) are shown the pre-processing resultant parameters for a welding experiment with an induced perturbation on the weld pool path (ferric chloride) as is shown in the Figure 7(c). By just looking the quality level 1 waveform (see Figure 7(a)), the perturbation interference is imperceptible, but when the quality level 2 is observed, it is possible to note some sudden variatiotly response considering variations and perturbations detected by each sns at the presence of the induced perturbation. Hence, which of the two quality level parameters is more accurate and reliable for assessing the welding quality? A data fusion process could give a more exactly response considering variations and perturbations detected by each sensor.

Before the application of data fusion concept, it is necessary modeling the quality level signals. Each signal could be considerate as time series and it is modeled as a parametric model:

(13) $${\text{X}}_{\text{t}+1}={\text{F}}_{\text{t}+1}{\text{X}}_{\text{t}}+{\text{w}}_{\text{t}}$$

where t is time instant, F_t+1_ is the transition matrix from the state X_t_ to X_t+1_, X in this work, is the quality level parameter and W is the noise, represented as a random variable with normal distribution with zero mean and variance Q.

The model for the quality level measurer is shown in Equation (14):

(14) $${\text{m}}_{\text{t}}={\text{x}}_{\text{t}}+{\text{v}}_{\text{t}}$$

The signal given by the sensor (m_t_) is the quality level measured by each arc emission sensor (X_t_) with an added noise (V_t_ = V_ex_ + V_in_) The noise also is a random variable with normal distribution with zero mean, but variance Q. With this model it is performed an overall quality assessment system by data fusion.

### 3.2. Data Fusion

Data fusion is the process of combining and integrating measured features originated from different sensors to produce more specific, comprehensive, and unified information about a monitored process such as the arc welding features in the case of this paper. Diverse parameters are involved in many production processes. Measuring their behavior is very important for achieving a high quality production. Some measured parameters m_t_ are used by workers for visualization (or settings) of the production line and other parameters are used as feedback variables for process control systems. The measurement systems are composed of transducers and sensors which measure and read diverse variables and parameters of production processes. Along with signals of parameters, X_t_ undesirable noise signals V_ex_ are also measured (external noise) and both signals are altered by circuits of conditioning, transmission and calibration; These signal management circuits also adds V_in_ noise on the measured signals (internal noise). The external noise V_ex_ has intrinsic nature in the processes as well as the internal noise V_in_ in the measurement systems and both signals V_ex_+V_in_ are responsible for the errors of measurement. Finally, the measured signal m_t_ is constituted of three components: the measured variable, external noise and internal noise (m_t_= X_t_ + V_ex_ + V_in_).

Data fusion in sensing (named also as multi-sensor fusion) are a set of techniques broadly used in science areas such as image processing, remote sensing, sensor networks, *etc*. The goal of these techniques is to retrieve and to synthesize data from numerous sources (variables and parameters from processes) [14]. Measurement systems based on single sensors are limited in robustness when the noise level increases (external and internal). Data fusion approach was used for welding quality assessment by NDT methods X-ray and ultrasonic techniques;. The interest of data fusion relies on the use of complementary methods: more information about the sample is thus available than with one method alone. Another interest of data fusion is the improvement of reliability by using the redundancy of the methods. This happens when several methods detect the same object with a rather low confidence, then, of course the data fusion presents a great advantage because the confidence after fusion increases [15]. Data fusion of welding parameters (welding speed, wire feed rate, arc voltage, contact-tube-to-work distance—CTWD, and weld pool width) also was approached to monitoring the geometry of the welding bead (depth of welding penetration) [16].

Multi-sensor measurement systems offer numerous advantages over single sensors when it comes to the fundamental tasks of utilizing and delivering information for a specific objective [14–18]. In the case of the GMAW-S process by using a specific sensor disturbance specifics can be drawn, showing the need of using more than only one sensor. There are different data fusion methods and one of them is the Kalman Filter—KF. In KF fusion method there are two broad approaches: measurement fusion and state-vector fusion. State-vector fusion is preferable in such practical situations [12]. In such a system, each sensor uses an estimator that obtains an estimate of the state vector and its associated covariance matrices from the data of that associated emission sensor. Then these state vectors are transmitted over a data link to the fusion center. As shown in Figure 7, state-vector fusion methods use a group of Kalman filters to obtain individual sensor-based state estimates which are then fused to obtain an improved joint state estimate. The KF is given for each set of observations, meaning that the algorithm is applied independently for each sensor (data) and generates state estimates.

State and covariance time propagation:

(15) $${\hat{\text{X}}}_{\text{t}}^{-}={\text{F}}_{\text{t},\text{t}-1}{\hat{\text{X}}}_{\text{t}-1}$$

(16) $${\text{p}}_{\text{t}}^{-}={\text{F}}_{\text{t},\text{t}-1}{\text{P}}_{\text{t}-1}{\text{F}}_{\text{t},\text{t}-1}^{\text{T}}+{\text{Q}}_{\text{t}-1}$$

State and covariance measurement update:

(17) $${\hat{\text{x}}}_{\text{t}}{=\hat{\text{x}}}_{\text{t}}^{-}{+\text{K}}_{\text{t}}\left({\text{y}}_{\text{t}}-{\text{H}}_{\text{t}}{\hat{\text{x}}}_{\text{t}}^{-}\right)$$

(18) Kt=Pt−HtT(H_tP_t^−+R_t)−1

(19) $${\text{P}}_{\text{t}}=\left(\text{I}-{\text{k}}_{\text{t}}{\text{H}}_{\text{t}}\right){\text{P}}_{\text{t}}^{-}$$

First, the state estimate is generated by processing the measurement data from each sensor. Fusion is obtained by combining the state estimates using a weighted sum of the two independent state estimates. The weight factors used are the appropriate covariance matrices. Thus, these state estimates and the corresponding covariance matrices are fused as follows, that is, the fused state and covariance matrix are computed using the following expressions:

(20) $${\hat{\text{X}}}_{\text{t}}^{\text{f}}={\hat{\text{X}}}_{\text{t}}^{1}+{\hat{\text{P}}}_{\text{t}}^{1}{\left({\hat{\text{P}}}_{\text{t}}^{1}+{\hat{\text{P}}}_{\text{t}}^{2}\right)}^{-1}\left({\hat{\text{X}}}_{\text{t}}^{1}-{\hat{\text{X}}}_{\text{t}}^{2}\right)$$

(21) $${\hat{\text{P}}}_{\text{t}}^{\text{f}}{=\hat{\text{P}}}_{\text{t}}^{1}-{\hat{\text{P}}}_{\text{t}}^{1}{\left({\hat{\text{P}}}_{\text{t}}^{1}+{\hat{\text{P}}}_{\text{t}}^{2}\right)}^{-1}{\hat{\text{P}}}_{\text{t}}^{1^{\text{T}}}$$

**Figure 8.**
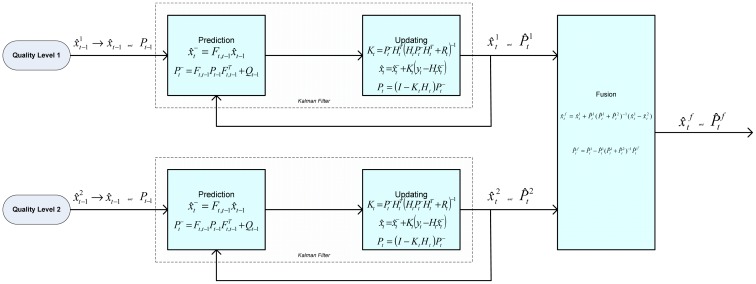
Detailed data fusion architecture.

Here, ${\hat{\text{X}}}_{\text{t}}^{1}$ and ${\hat{\text{X}}}_{\text{t}}^{2}$ are the estimated state vectors of filters 1 and 2 with measurements from sensor 1 and sensor 2, respectively, and ${\hat{\text{p}}}_{\text{t}}^{1}$ and ${\hat{\text{p}}}_{\text{t}}^{2}$ are the corresponding estimated state error covariance from filters 1 and 2. Figure 8 shows the overall data fusion architecture.

### 3.3. Quality Assessment

**Figure 9.**
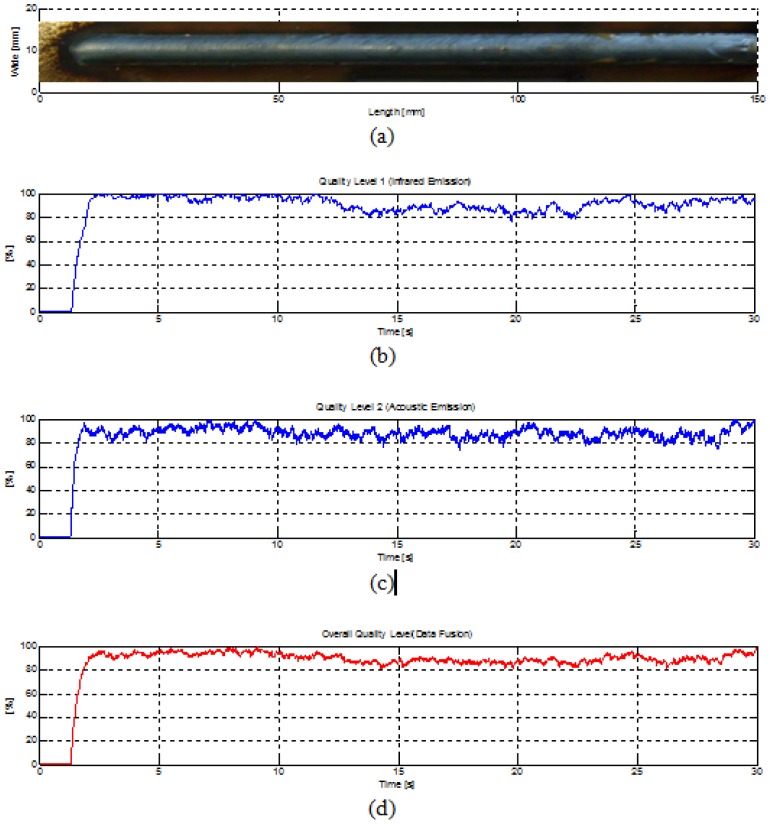
Quality level parameters, (**a**) welding trial with induced perturbation, (**b**) From infrared signal, (**c**) From acoustical signal, (**d**) overall quality measured based on data fusion.

Figure 9(a) shows a welding experiment with an induced perturbation (paint on the welding pool path). In Figure 9(b,c) the measured quality levels are shown. The perturbation presence is notorious in the first parameter, but it is not noticed clearly in the second parameter. In Figure 9(d) the data fusion resultant that accuses the presence of some perturbation is shown. It is clear that this system has limitations for showing notoriety when there is some perturbation and it could become unreliable, but its principal advantage is that this system has more capability to detect perturbations that other quality assessment systems based on measurements done just for one sensor. The quality level parameters were modeled as a time series following a parametric model and after numerous experiments, signatures of average and standard deviation were obtained and they are used as comparison reference in the pre-processing stage to measure the quality level of either sensor. This fact also is a high limitation of this system; a model of the quality in welding could complement the present work eliminating the dependence of certain constants on our quality assessment rule that they are obtained experimentally.

## 4. Conclusions

A quality assessment system based on monitoring of arc welding emissions and data fusion was performed. The data fusion process has shown positive results detecting induced perturbations throughout the welding path in comparison at usual quality assessment methods based on single sensoring. Many researchers obtain quality level models as a time series or mechanistic models becoming the quality assessment system dependent on some constants that are usually obtained experimentally, which makes the assessment system unreliable. This limitation is related to the lack of relationships between welding quality models and the welding parameters and this drawback is avoided by using multiple sensoring techniques. By using data fusion of quality levels, the capability and sensitive of the overall quality assessment system were improved.

By monitoring arc welding emissions it was possible to detect induced perturbations during the welding runs. Some perturbations are detected by the acoustic emissions and others by infrared emissions. Acoustic monitoring was sensitive to environmental noise and the quality level extracted from it, has higher ripples than the quality level sensed through infrared emissions. Sensoring based on data fusion improves the monitoring of the welding quality and it could be an alternative to classical on-line methods of assessment and inspection used for detecting and finding disturbances that are based in direct measurements of parameters such as arc voltage, welding current, wire feed speed, and others.
